# Predifferentiated amniotic fluid mesenchymal stem cells enhance lung alveolar epithelium regeneration and reverse elastase-induced pulmonary emphysema

**DOI:** 10.1186/s13287-019-1282-1

**Published:** 2019-06-13

**Authors:** Ying-Wei Lan, Jing-Chan Yang, Chih-Ching Yen, Tsung-Teng Huang, Ying-Cheng Chen, Hsiao-Ling Chen, Kowit-Yu Chong, Chuan-Mu Chen

**Affiliations:** 1 Department of Life Sciences, College of Life Sciences, National Chung Hsing University, No. 250, Kuo Kuang Rd., Taichung, 402 Taiwan; 2 Department of Medical Biotechnology and Laboratory Science, College of Medicine, Chang Gung University, Taoyuan, 333 Taiwan; 3 Graduate Institute of Biomedical Sciences, Division of Biotechnology, College of Medicine, Chang Gung University, Taoyuan, 333 Taiwan; 4 Department of Internal Medicine, China Medical University Hospital, Taichung, 404 Taiwan; 5 College of Health Care, China Medical University, Taichung, 404 Taiwan; 6 Department of Bioresource, Da-Yeh University, Changhwa, 515 Taiwan; 7 Department of Laboratory Medicine, Chang Gung Memorial Hospital, Linkou, Taoyuan, 333 Taiwan; 8 Centre for Stem Cell Research, Faculty of Medicine and Health Sciences, Universiti Tunku Abdul Rahman, 43000 Kajang, Selangor Malaysia; 9 The iEGG and Animal Biotechnology Center, and Rong Hsing Research Center for Translational Medicine, National Chung Hsing University, Taichung, 402 Taiwan

**Keywords:** Predifferentiation, Amniotic fluid mesenchymal stem cell, Elastase-induced pulmonary emphysema

## Abstract

**Introduction:**

Pulmonary emphysema is a major component of chronic obstructive pulmonary disease (COPD). Emphysema progression attributed not only to alveolar structure loss and pulmonary regeneration impairment, but also to excessive inflammatory response, proteolytic and anti-proteolytic activity imbalance, lung epithelial cells apoptosis, and abnormal lung remodeling. To ameliorate lung damage with higher efficiency in lung tissue engineering and cell therapy, pre-differentiating graft cells into more restricted cell types before transplantation could enhance their ability to anatomically and functionally integrate into damaged lung. In this study, we aimed to evaluate the regenerative and repair ability of lung alveolar epithelium in emphysema model by using lung epithelial progenitors which pre-differentiated from amniotic fluid mesenchymal stem cells (AFMSCs).

**Methods:**

Pre-differentiation of eGFP-expressing AFMSCs to lung epithelial progenitor-like cells (LEPLCs) was established under a modified small airway growth media (mSAGM) for 7-day induction. Pre-differentiated AFMSCs were intratracheally injected into porcine pancreatic elastase (PPE)-induced emphysema mice at day 14, and then inflammatory-, fibrotic-, and emphysema-related indices and pathological changes were assessed at 6 weeks after PPE administration.

**Results:**

An optimal LEPLCs pre-differentiation condition has been achieved, which resulted in a yield of approximately 20% lung epithelial progenitors-like cells from AFMSCs in a 7-day period. In PPE-induced emphysema mice, transplantation of LEPLCs significantly improved regeneration of lung tissues through integrating into the lung alveolar structure, relieved airway inflammation, increased expression of growth factors such as vascular endothelial growth factor (VEGF), and reduced matrix metalloproteinases and lung remodeling factors when compared with mice injected with AFMSCs. Histopathologic examination observed a significant amelioration in DNA damage in alveolar cells, detected by terminal deoxynucleotidyltransferase-mediated dUTP nick end labeling (TUNEL), the mean linear intercept, and the collagen deposition in the LEPLC-transplanted groups.

**Conclusion:**

Transplantation of predifferentiated AFMSCs through intratracheal injection showed better alveolar regeneration and reverse elastase-induced pulmonary emphysema in PPE-induced pulmonary emphysema mice.

**Electronic supplementary material:**

The online version of this article (10.1186/s13287-019-1282-1) contains supplementary material, which is available to authorized users.

## Background

Chronic obstructive pulmonary disease (COPD) is characterized by airflow limitation and irreversible lung structure damage, leading to steadily increasing mortality rates [1]. Emphysema is one of the major components of COPD, which is characterized by persistent and chronic inflammation, alveolar walls destruction, and permanent enlargement of air spaces. This leads to progressive disability and death in COPD patients worldwide [2].

Inhalation of noxious substances, such as cigarette smoke, is the major risk factor for developing emphysema. Years of noxious particle exposure causes the infiltration of inflammatory cells, especially neutrophils and macrophages, into the airways. This results in the activation of various proteolytic enzymes and proteinases, including neutrophil elastase and matrix metalloproteinase-9, which destroy the alveolar structure [3]. Apoptosis of lung epithelial and endothelial cells is critical for the pathogenesis of emphysema caused by cigarette smoke exposure [4]. Cigarette smoke exposure can progressively reduce antioxidant and autophagic defensive abilities in lung epithelial and endothelial cells, thereby driving cells towards apoptosis [5]. Persistent oxidative stress could deplete the balance between self-renewal and cell differentiation in stem cells and progenitors, which results in impaired alveolar regeneration in lung tissue with emphysema [6].

Current treatments for COPD include reduction of dyspnea, prevention of exacerbation and disease progression, and improvement of quality of life to reduce mortality [7]. After decades of efforts to identify therapeutic strategies to treat COPD, smoking cessation and anti-inflammatory pharmacologic agents, such as antagonists of cytokines such as tumor necrosis factor-α and interleukin-8, are the mainstays of treatment [8]. However, under these therapeutic approaches, while COPD patients do improve their life quality and reduce both symptoms and acute exacerbations, there is no prevention of disease progression and therefore no reduction in mortality [9]. Stem and progenitor cell therapies have been widely considered as the best potential candidates for the treatment of respiratory diseases and destructive disorders such as COPD/emphysema [9, 10]. Their capability of differentiating into various cell types [11], immunomodulatory effects [3], paracrine effects [12], and anti-apoptosis [13] can help to repair and regenerate lung tissue after injury.

Various stem cells and delivery routes have been used to treat experimental models of elastase-induced emphysema [5]. Transplantation of embryonic stem cells (ESCs) that can be differentiated to type II alveolar epithelial cells have been shown to improve symptoms in the mouse and patient with emphysema [14, 15]. Other sources of MSCs harvested from various tissues (including bone marrow, adipose, umbilical cord blood, amniotic fluid and lung tissue) revealed some beneficial effects on the reversal of lung structure destruction, tissue regeneration, neutrophil infiltration, and collagen deposition [9, 16].

Although transplanted stem or progenitor cells contribute only marginally to lung regeneration, a variety of microenvironmental mediators in the damaged lung can affect precisely differentiation of engrafted cells. This may influence the therapeutic efficacy for the repair of the damaged lung. Thus, in other disease models, such as neurological disease and myocardial infarction, predifferentiating stem cells into specific cell types prior to transplant might be a better approach [17, 18]. Direct transplantation of type II alveolar epithelial cells, which are known progenitor cells for alveolar epithelium, also modulates the inflammatory response and alveolar edema fluid clearance. This is accomplished by secretion of soluble paracrine factors, and alveolar epithelial barrier restoration occurs via differentiation into type I alveolar epithelial cells in acute lung injury [19, 20] and fibrotic animal models [21, 22]. Similarly, established stable hESCs or induced pluripotent stem cells (iPSCs) that can be differentiated and enriched into a pure population of type II alveolar epithelial cells may be suitable for transplantation to treat lung injury [23, 24].

AFMSCs are particularly interesting because of their potential benefits when considering them for medical use. Amniotic fluid is a novel stem cell source that is regarded as medical waste. AFMSCs have the potential to differentiate into each of the embryonic germ layer cells, remarkable self-renewal ability, and a lack of ethical concern and are easy to isolate and abundantly available, and they possess privileged immunological characteristics that make them an ideal and reliable candidate for cell therapy [25]. Several papers have reported that engrafted AFMSCs can differentiate into lung lineage cells and integrate into damage sites or induce local regeneration in lung injury animal models [16, 26, 27].

In this work, we aim to identify if predifferentiation of AFMSCs to LEPLCs before transplantation would result in a better outcome in terms of cell survival, anti-inflammatory response, tissue regeneration, and histopathological improvement in a lung emphysema animal model.

## Methods

### Isolation and characterization of murine AFMSCs

AFMSCs were derived from the amniotic fluid of pregnant eGFP-transgenic mice (days 11.5). First, the yolk sac was ruptured with a 27-gauge needle. AFMSCs were recovered and cultured in α-minimal essential medium (α-MEM) (Life Technologies) supplemented with 10% FBS and 1% penicillin/streptomycin and were incubated at 37 °C in a 5% CO_2_ incubator as described in Wen et al. [28] (Additional file 2: Figures S1A and S1B).

### Flow cytometry analysis

Flow cytometry was used to examine the purity of isolated AFMSCs by detection CD44, stem cell antigen 1 (Sca-1), CD105 (eBioscience), CD34, CD90 (BD Biosciences), CD29, CD11b, CD73, CD106, and CD45 (R&D Systems) expression on the cell surface as described in Peng et al. [29] (Additional file 2: Figure S1E). To confirm the percentage of differentiated AFMSCs towards LEPLCs, we detect the presence of TTF-1, SP-C, AQP-5, and CCSP in cells according to the manufacturer’s instructions. Briefly, detached cells were fixed with 4% paraformaldehyde for 15 min, permeabilized with 0.25% Triton X-100 for 15 min, blocked with 4% BSA in PBS for 1 h, and then incubated overnight at 4 °C with the following intracellular marker antibodies at appropriate dilutions: TTF-1 (Abcam, 1:250 dilution), SP-C, AQP-5, and CCSP (Millipore, all at 1:250 dilutions). The cells were then incubated with an appropriate Alexa Fluor® 546 dye-conjugated secondary antibody (1:200) at room temperature for 2 h. After rinsing the cells twice, fluorescence was detected and analyzed using flow cytometry (FACSCalibur).

### In vitro differentiation

To induce the isolated AFMSCs towards differentiation into adipogenic and osteogenic cell lineages, cells were cultured to 100% confluency and then cultured in either an adipogenic medium (α-MEM containing 10% FBS, 1 μM dexamethasone, 0.5 mM isobutylmethylxanthine, 10 μg/mL insulin, and 100 μM indomethacin), or an osteogenic medium (α-MEM containing 10% FBS, 1 μM dexamethasone, 10 mM glycerol 2-phosphate, and 50 μM ascorbic acid 2-phosphate). The medium was changed twice a week. After 3 weeks of induction, Oil Red O staining was performed to evaluate the adipogenesis efficiency by examining the intracellular accumulation of lipid droplets (Additional file 2: Figure S1C). Additionally, alizarin red staining was performed to evaluate the osteogenesis efficiency by detecting calcium mineralization (Additional file 2: Figure S1D).

To induce the isolated AFMSCs towards differentiation into lung epithelial progenitor-like cells, we use optimal media formulation described previously, with slight modifications [30]. AFMSCs were plated on 0.1% gelatin-coated culture dishes for 24 h and cultured to confluency. Then, the culture medium was changed to, which comprises a small airway basal medium (SABM) supplemented with 0.5 mg/mL BSA, 5 mg/mL insulin, 10 mg/mL transferrin, 30 mg/mL bovine pituitary extract, 0.5 mg/mL epinephrine, 0.5 mg/mL hydrocortisone, 0.5 ng/mL human EGF, 1% penicillin-streptomycin, and 50 ng/mL FGF-10 [30]. The medium was changed every 2 days. After 7 days of induction, flow cytometry analysis was performed to detect lung epithelial cell lineage markers.

### Intratracheal transplantation of stem cells into PPE-induced lung emphysema murine model

Pulmonary emphysema was induced by intratracheal instillation, as previously described [12, 31]. Eight-week-old male ICR mice were purchased from the Lasco (Taipei, Taiwan). All experimental procedures were approved by the Institutional Animal Care and Use Committee of Chang Gung University (Taoyuan, Taiwan; IACUC No. CGU15-156), and the experiments were performed in accordance with the guidelines. Mice were randomly picked to different groups, and there were at least 5 or more mice in each group. Each mouse was intratracheally administered 1.5 mg/kg porcine pancreatic elastase (PPE) dissolved in 50 μL sterile PBS on day 0. After 2-week period of PPE treatment, mice were randomly selected for intratracheal injection (1 × 10^5^ cells in 50 μl PBS) of AFMSCs, LEPLCs, or PBS. After 4-week period of stem cells/PBS treatment, the mice were sacrificed by an overdose of 2.5% avertin and the therapeutic effects were examined.

### Histology and immunofluorescence (IF)

Left lung tissues were fixed with 10% formalin overnight and paraffin embedded. Tissues were sectioned and stained with hematoxylin and eosin (H&E) according to standard protocols [32]. The severity of lung emphysema was assessed by measuring the mean linear intercept (Lm) [33]. The distance in airspace size between the alveolar walls was calculated by drawing equally distributed horizontal lines across each alveolus from wall to wall and recording the length for each measurement. Five random microscopic fields within each lung section were observed [34]. For IF analysis, the paraffin-embedded sections were deparaffinized and then blocked with 4% fetal bovine serum in PBS with 0.2% Triton X-100 for 2 h at room temperature. Sections were then incubated overnight at 4 °C with primary antibody as follows: anti-pro-SPC (Millipore, 1:4000), anti-AQP-5 (Millipore,1:800), and anti-GFP (Millipore, 1:750). Sections were incubated with secondary antibody (Alexa Fluor® 488 conjugated goat anti-mouse IgG or Alexa Fluor® 647 conjugated goat anti-rabbit IgG) for 1 h at RT. After washing with PBS, the slides were stained with DAPI for nuclear counterstaining and mounted with FluoreGuard Mounting Medium (Biosystems).

### Masson trichrome and Sirius red stain for lung fibrosis analysis

To determine collagen content, the lung tissue sections were subjected to Masson’s Trichrome staining (TRM-2, SCYTEK Laboratories) according to the manufacturer’s protocol. For Picrosirius red staining, slides were deparaffinized and rehydrated and then stained with hematoxylin for 1 min. After washing, the slide was immersed in Picrosirius red solution for 30 min. Then, the slides were washed in 4% Glacial acetic acid solution, dehydrated, and mounted. Stained slides were examined using bright field microscopy.

### Terminal deoxynucleotidyl transferase dUTP nick-end labeling staining (TUNEL) assay

To detect the apoptotic cells in the damaged lung, lung tissue sections were subjected to TUNEL staining using the In Situ Cell Death Detection Kit, Fluorescein (Roche) according to the manufacturer’s protocol. The stained slides were then stained with DAPI for nuclear counterstaining and mounted with FluoreGuard Mounting Medium.

### RNA isolation and quantitative real-time RT-PCR

Total RNA was prepared from lung tissue using the EasyPrep Total RNA Kit (TOOLS). RNAs were reverse transcribed into cDNAs using an MMLV Reverse Transcription kit (Protech). Quantitative real-time RT-PCR was performed using LightCycler 480 SyberGreen I Master Mix and the LightCycler® 480 Instrument (Roche) as previously described [35]. Sequences of the gene-specific primers used are listed in Additional file 1: Table S1. Relative gene expression was determined by the ∆∆Ct method, where Ct is the threshold cycle. The relative mRNA expression levels were normalized to the mRNA level of the reference *Gapdh* gene.

### Western blot analysis

Western blot analysis to examine the indicated proteins was performed as described previously [36]. Brief, 50 μg of total proteins from cell lysates was loaded onto each lane and the proteins were separated in sodium dodecyl sulfate polyacrylamide electrophoresis (SDS-PAGE; Bio-Rad Laboratories). After electrophoresis, the resolved proteins were transferred to PVDF membrane (Millipore). The membranes were blocked with 5% skimmed milk powder (Anchor) in phosphate-buffered saline-Tween (PBS-T): phosphate-buffered saline (PBS, Sigma-Aldrich) containing 0.1% Tween-20 in (Sigma-Aldrich) for 2 h and probed overnight with the following antisera at appropriate dilutions: 1:500 dilution of the anti-proSPC and anti-AQP-5 (Millipore) and a 1:10,000 dilution of the anti-β-actin (Novus Biologicals) antisera in PBS-T. Identification of each protein was achieved with the Western Lightning ECL Plus (Millipore) using an appropriate horseradish peroxidase (HRP)-conjugated secondary antibodies (Jackson Immuno Research Laboratories). Protein levels in the western blot analysis were detected and quantified by the Amersham Imager 600 imaging system (GE Healthcare Life Sciences). To adjust for loading differences, the optical density of each protein was normalized to that of the β-actin band.

### Statistical analysis

Data are presented in bar graphs as the mean ± SD. Differences between groups were analyzed using one-way analysis of variance analysis (ANOVA), followed by the Dunnett’s post hoc test. When results were not normally distributed, a Kruskal-Wallis test followed by Dunn’s tests between groups was performed. All data were plotted and analyzed using GraphPad Prism. For all analyses, a *p* value < 0.05 was considered statistically significant.

## Results

### Optimization of lung cell lineage differentiation in AFMSCs

**Fig. 1:**
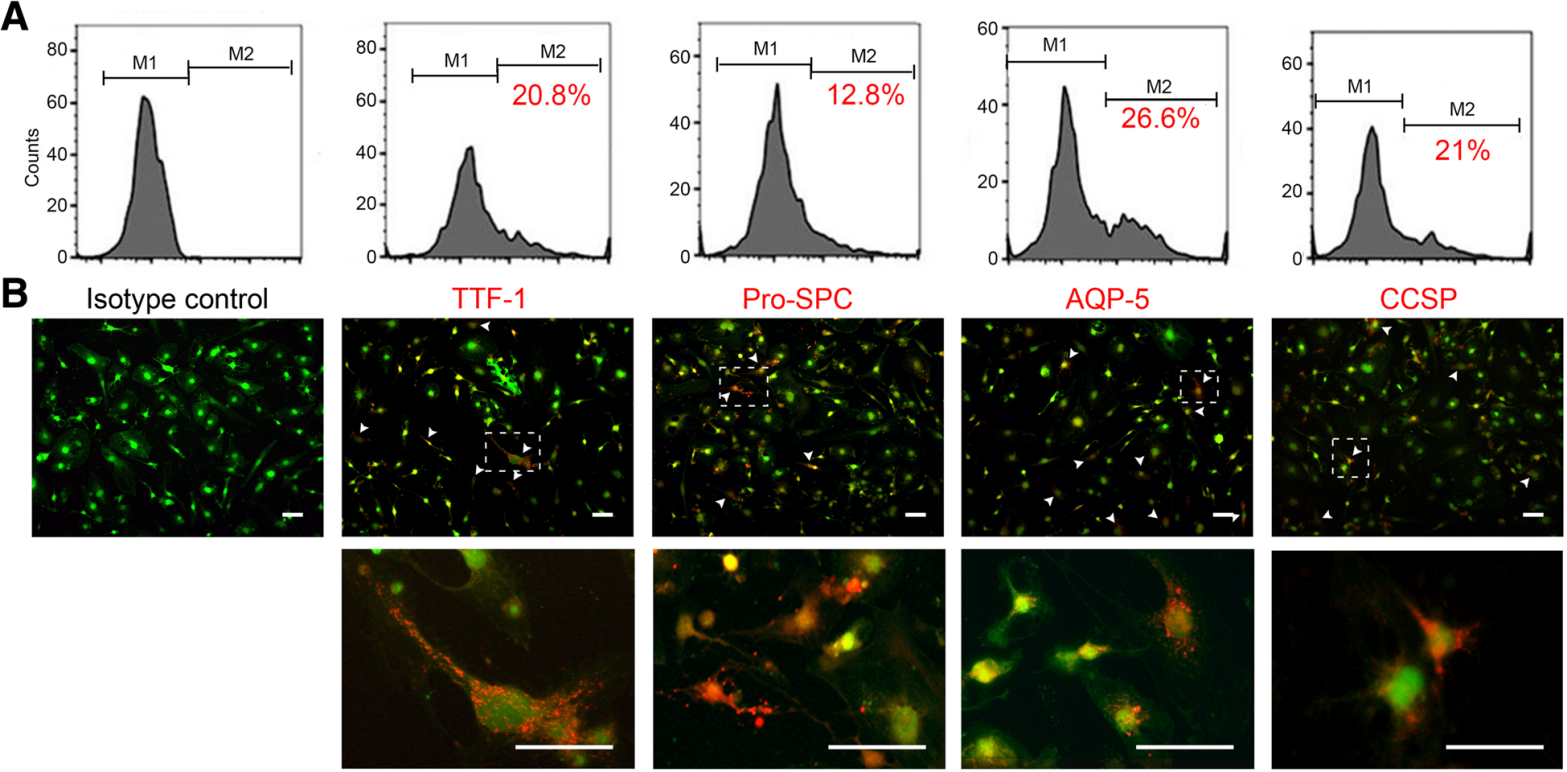
Characterization of predifferentiated AFMSCs (LEPLCs) under modified small airway growth medium (mSAGM) induction. AFMSCs were differentiated for 7 days and then immunostained with lung epithelial progenitor-like markers TTF-1, SPC, AQP-5, and CCSP. **a** Flow cytometry analysis was performed. **b** Immunocytochemistry staining for endogenous eGFP (green) and lung epithelial progenitor-like markers (red) in differentiated cells at 7 days. Scale bar = 200 μm

To induce differentiation into lung cell lineages, AFMSCs were cultured in a modified small airway growth media (mSAGM). This lead to the highest expression of surfactant protein C (SPC; a marker for type II alveolar epithelial cells) and thyroid transcription factor 1 (TTF-1, a marker for lung cell precursors and an essential regulator for a series of lung cells) expressing cells [30]. To decide the optimal differentiation condition, flow cytometry was used to analyze a series of lung epithelial markers in type I alveolar epithelial cells (aquaporin 5, AQP-5), type II alveolar epithelial cells (SPC), clara cells (clara cell secretory protein, CCSP), and lung precursor (TTF-1) at different time points. After 5 days of incubation, AFMSCs started to differentiate towards lung precursor (3.19% TTF-1^+^ cells) and lung cell lineages (2.13% SPC^+^, 3.14% AQP-5^+^, and 4% CCSP^+^ cells) (Additional file 3: Figure S2A). We then observed a marked increase in the percentage of the lung precursor (20.8% TTF-1^+^ cells) and lung cell lineages (12.8% SPC^+^, 26.6% AQP-5^+^, and 21% CCSP^+^ cells) at 7 days (Fig. 1a). At 9 days, more AFMSCs differentiate to lung precursor (36.5% TTF-1^+^ cells) and lung cell lineages (69.8% SPC^+^, 61% AQP-5^+^, and 27.6% CCSP^+^ cells) (Additional file 3: Figure S2B). Protein expression of these markers in the differentiated AFMSCs were then confirmed by immunofluorescence (Fig. 1b). Type I alveolar epithelial cells were characterized as the terminally differentiated nonproliferative state, and 9 days of incubation resulted in high AQP-5 expression. Therefore, a 7-day incubation was used for the following experiments.

### Downregulated expression of emphysema factors in LEPLC-transplanted PPE-induced pulmonary emphysema mice

**Fig. 2:**
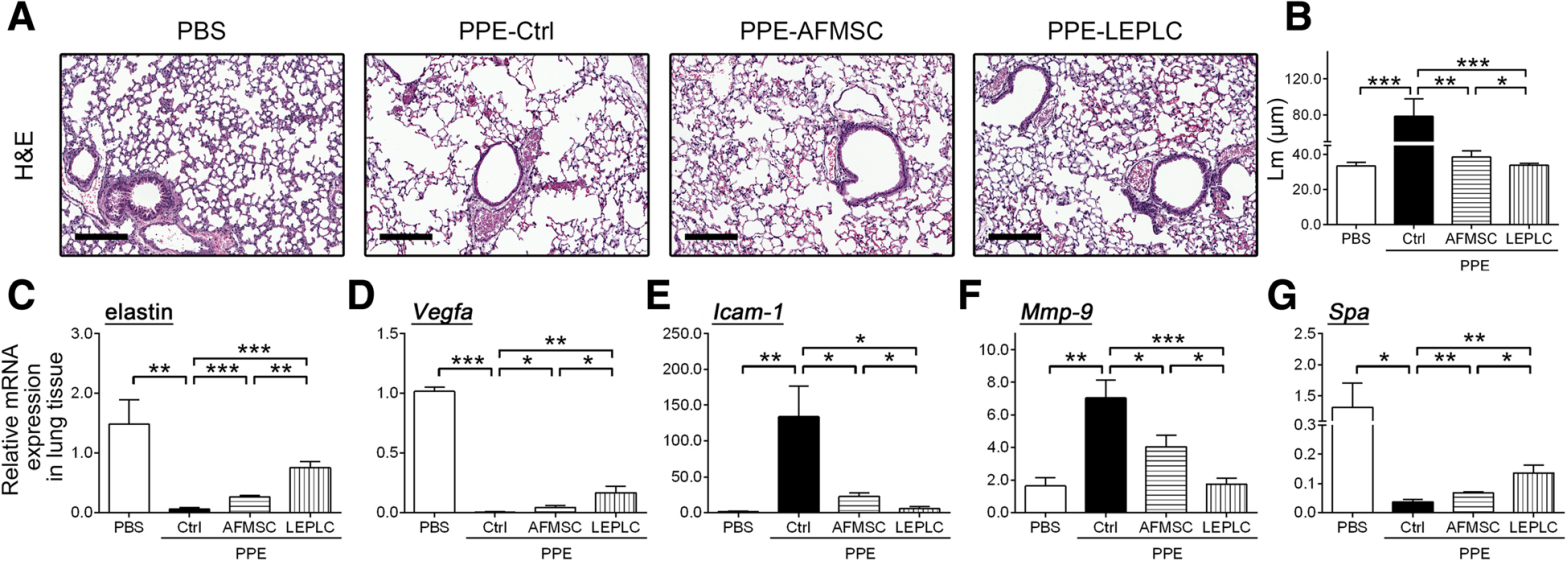
Transplantation of LEPLCs reduced emphysematous histopathologic changes and downregulated the expression of emphysema-related genes in the PPE-induced pulmonary emphysema mouse model. Effects of LEPLCs treatment on tissue regeneration and reversal of lung emphysema. Lung tissues were collected at week 6 post-PPE treatment. **a** H&E stained histological sections from each group. Scale bar = 100 μm. **b** H&E staining was calculated as histopathological scores, *n* ≥ 5. Quantitative real-time RT-PCR was performed to analyze the mRNA expression levels of emphysema-related genes, **c** elastin, **d**, *Vegfa*, **e** *Icam-1*, **f** *Mmp-9*, and **g** *Spa*. Values were normalized to the glyceraldehyde-3-phosphate dehydrogenase (*Gapdh*) gene and expressed in relation to the PBS group. Data are represented as the mean ± SD; *n* ≥ 5 per group. *, *p* < 0.05; **, *p* < 0.01; ***, *p* < 0.001

To assess the effects of LEPLCs in the PPE-induced pulmonary emphysema model, the mRNA expression levels of candidate emphysema-related genes were determined. We examined the expression of the extracellular matrix gene which assembled normal pulmonary architecture, elastin [37], the cell adhesion molecule, which recruits inflammatory cells to the damaged lung, intercellular adhesion molecule 1 (*Icam-1*) [37], the protease, and matrix metalloproteinase-9 (*Mmp-9*) [38]; the mediator that maintain the homeostasis of alveolar compartment, vascular endothelial growth factor a (*Vegfa*) [39]; and mediator secreted specifically by type II alveolar epithelial cells which can reduce alveoli surface tension, surfactant protein A (*Spa*) [16]. Downregulation of elastin, *Vegfa*, and *Spa* mRNA levels and upregulation of *Icam-1* and *Mmp-9* were detected after 6 weeks of PPE treatment when compared with the PBS control group (Fig. 2c to g). Although AFMSCs transplantation increased elastin and *Vegfa* mRNA levels and reduced *Icam-1* and *Mmp-9* mRNA levels, there was a more significant improvement in the LEPLCs transplantation group at 4-weeks after stem cell transplantation (Fig. 2c to g).

We next investigated the lung histopathologic sections from each experimental group at 4 weeks post stem cell transplantation (Fig. 2a). H&E staining showed that alveolar space increased in the lungs injected with PPE but was significantly attenuated by transplantation of AFMSCs and LEPLCs (Fig. 2a). Quantification of alveolar destruction was measured using the mean linear intercept length (Lm). The Lm was markedly increased in the PPE group when compared to the PBS control group, and the Lm was decreased in the AFMSC-transplanted mice and was further decreased in the LEPLC-transplanted mice (Fig. 2b).

### Downregulated expression of inflammatory and fibrotic factors in LEPLC-transplanted PPE-induced pulmonary emphysema mice

**Fig. 3:**
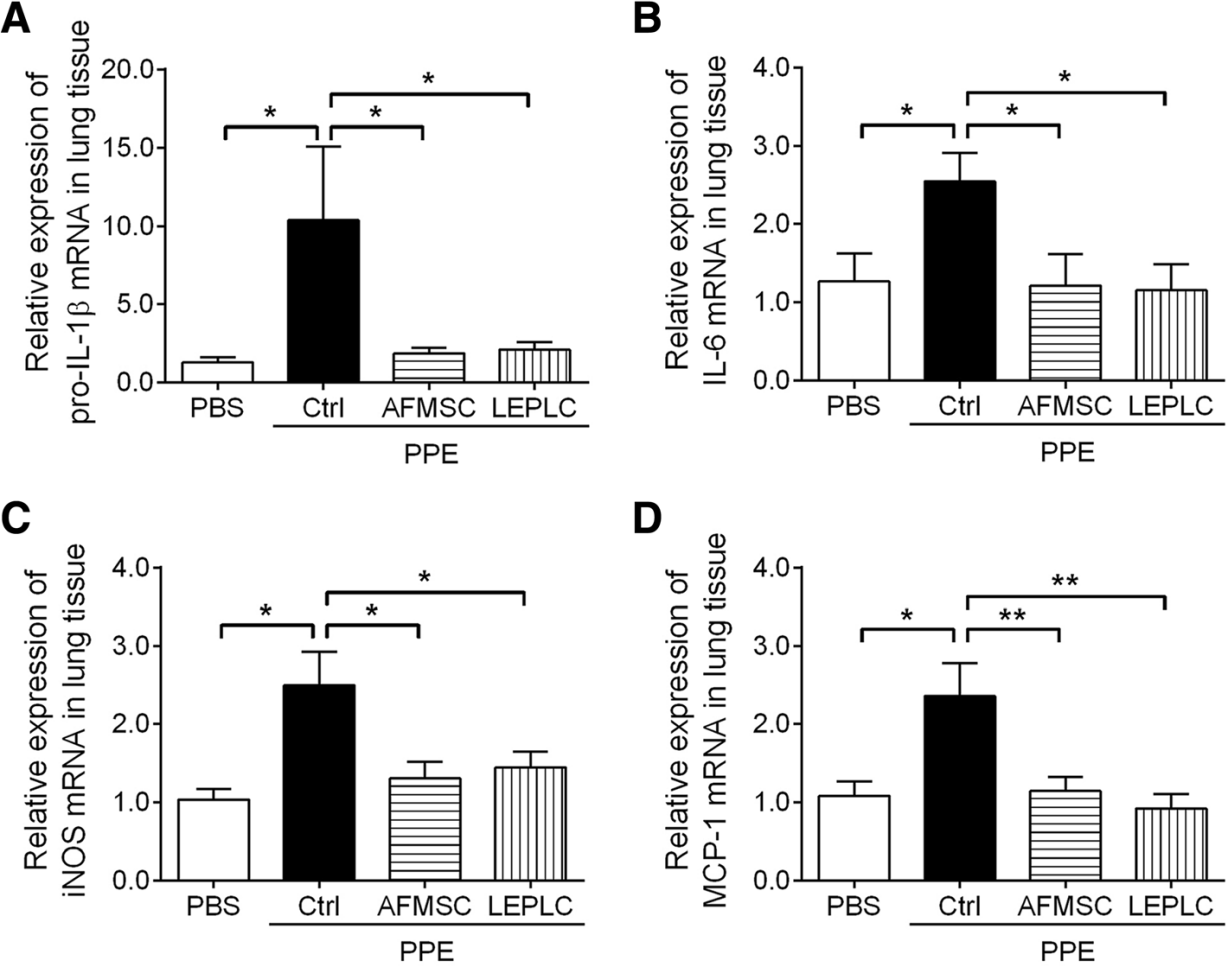
Transplantation of LEPLCs downregulated the expression of inflammatory mediators in the PPE-induced pulmonary emphysema mouse model. Effects of LEPLCs treatment on the inflammatory response. Lung tissues were collected at week 6 post-PPE treatment for inflammatory-related genes, and mRNA expression levels of **a** *pro-Il-1β*, **b**, *Il-6*, **c** *Inos*, and **d** *Mcp-1* were determined by quantitative real-time RT-PCR. Values were normalized to the *Gapdh* gene and expressed in relation to the PBS group. Data are represented as the mean ± SD; *n* ≥ 5 per group. *p < 0.05; **p < 0.01

**Fig. 4:**
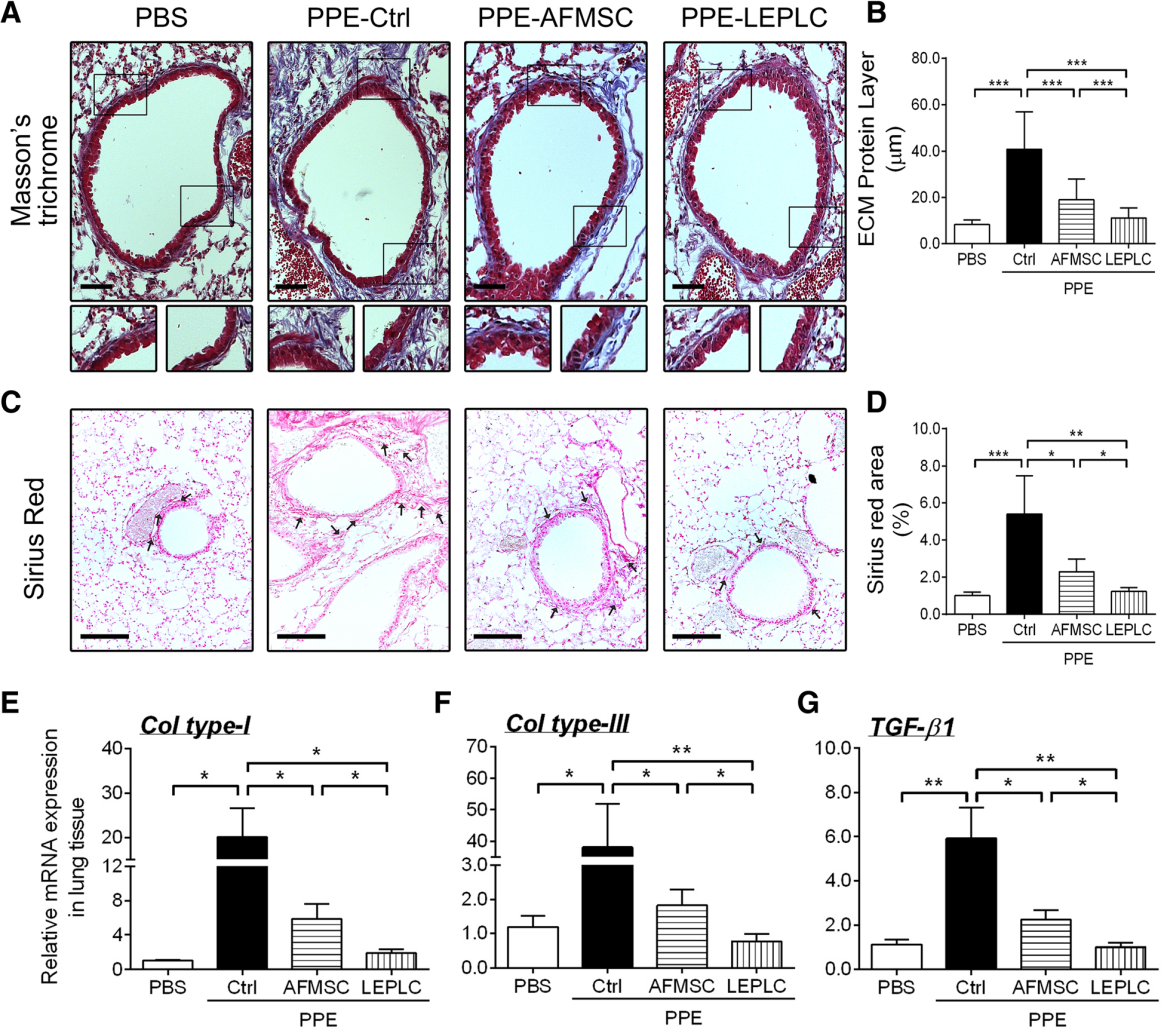
Transplantation of LEPLCs reduced fibrotic histopathologic changes and downregulated the expression of fibrotic-related genes in the PPE-induced pulmonary emphysema mouse model. Effects of LEPLCs treatment on extracellular matrix production and fibrosis-related genes. Lung tissues were collected at week 6 post-PPE treatment. **a** Masson’s trichrome, and **c** Sirius red stained histological sections from each group. Scale bar = 100 μm. The boxed regions in the **a** upper panel are magnified in the lower panel, and the violet staining indicates collagenous material. The fibrillar collagen appears as red structures (arrow). **b**, **d** Masson trichrome and Sirius red staining were calculated as histopathological scores. In all sub figs., *n* ≥ 5. Quantitative real-time RT-PCR was performed to analyze the mRNA expression of fibrotic-related genes **e** *ColI*, **f**, *ColIII*, and **g** *Tgf-β1*. Values were normalized to the *Gapdh* gene and expressed in relation to the PBS group. Data are represented as the mean ± SD; *n* ≥ 5 per group. * *p* < 0.05; ** *p* < 0.01

To assess the effects of LEPLCs on inflammation and fibrosis in the PPE-induced pulmonary emphysema model, mRNA expression levels of pro-inflammatory cytokine, pro-*Il-1β*, the inflammation-mediator, *Il-6*, inducible nitric oxide synthase (*Inos*), and monocyte chemotactic protein-1 (*Mcp-1*) were examined. Expression levels were significantly upregulated after 6 weeks of PPE treatment when compared with the PBS control group (Fig. 3a to d). Both AFMSCs and LEPLCs transplantation reduced these inflammatory mediators at 4 weeks after stem cell transplantation (Fig. 3a to d). Then, we determined the effect of LEPLCs on the expression of extracellular matrix components involved in the progression of lung fibrosis, including collagen types I and III, and transforming growth factor-beta 1 (*Tgf-β1*) (Fig. 4e to g). Expression of these fibrotic-related factors was markedly upregulated after 6 weeks of PPE treatment when compared with the PBS control group, but the mRNA levels were significantly reduced in the AFMSC-transplanted mice and further decreased in the LEPLC-transplanted mice (Fig. 4e to g). Pulmonary fibrosis is characterized by excessive extracellular matrix production and deposition; therefore, we performed Masson’s trichrome and Sirius red staining on lung histopathologic sections from each group after 4 weeks after stem cell transplantation (Fig. 4a and c). The thickness of the violet ECM layer around the small airways was significantly increased at 6 weeks post-PPE injection and was significantly reduced in the AFMSC-transplanted mice and further reduced in the LEPLC-transplanted mice (Fig. 4b). Deposition of collagen fibers was also examined by Sirius red staining, and a 6-fold increase in the amount of collagen fibers in the PPE group was detected. Transplantation of AFMSCs showed a significant decrease in the accumulation of collagen fibers, and we observed further reduction of collagen in the LEPLCs-transplantation group (Fig. 4d).

### Potential roles of transplanted LEPLCs in PPE-induced pulmonary emphysema mice

**Fig. 5:**
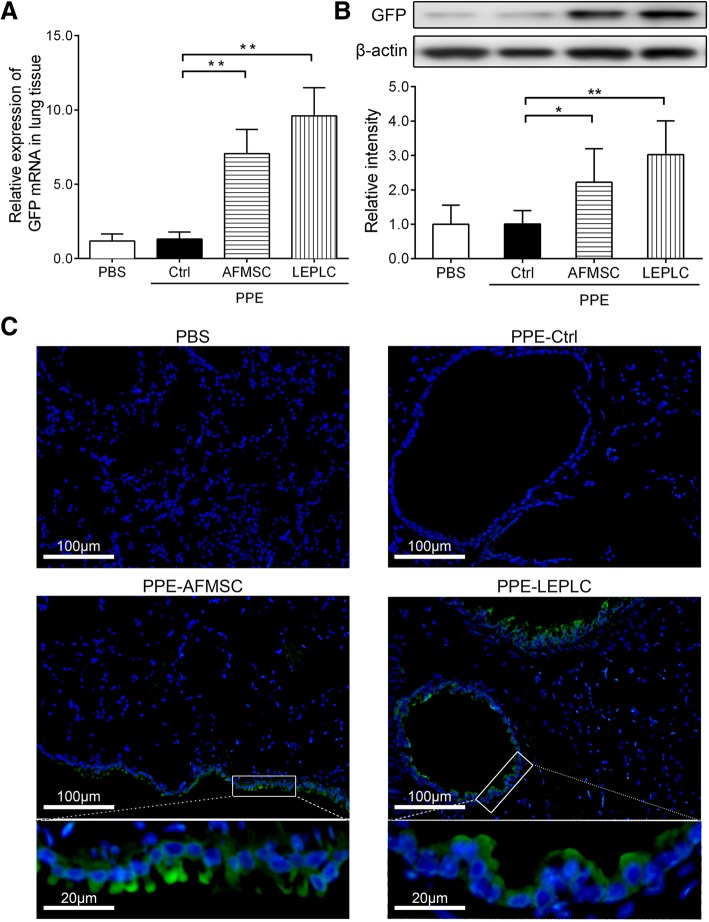
Expression and localization of eGFP-labeled transplanted cells in the lung of PPE-induced pulmonary emphysema mouse model. **a** Quantitative real-time RT-PCR and **b** Western blot analysis were performed to detect GFP expression in the lung tissues of mice after 4-week period of PBS, AFMSCs, or LEPLCs treatment following PPE administration. Values were normalized to *Gapdh* or β-actin levels and were expressed relative to the PBS group. *p < 0.05, **p < 0.01, and ***p < 0.001. Each dot represents an individual mouse with the mean shown for *n* > 5 per group. **c** Immunofluorescence staining for the distribution of GFP in lung tissues. Green signal observed in the lung section indicates GFP-positive cells and nuclei were stained with DAPI (blue). Magnified views with 20 μm bars (third row) expand on the boxed regions in the low-magnified images with 100 μm bars (second row)

To assess whether the engrafted LEPLCs trapped in and ameliorated the damaged lungs at 6 weeks post-PPE treatment, GFP-labeled AFMSCs or LEPLCs were intratracheally administered into the PPE-treated mice. Quantitative real-time RT-PCR showed an approximately 7–9-fold increase in *Gfp* expression levels, and Western blot analysis also showed an approximately 2–3-fold increase in both the AFMSC- or LEPLC-transplanted mice, but no GFP expression was detected in either the PBS or PPE-Ctrl groups (Fig. 5a and b). Immunofluorescence staining showed no GFP-labeled cells in lung tissue of the PBS or PPE-Ctrl groups, but numerous GFP-labeled cells were observed in both AFMSC or LEPLC treatment groups 4 weeks post stem cell transplantation (Fig. 5c). Most of the GFP-positive cells were distributed around the bronchi.

**Fig. 6:**
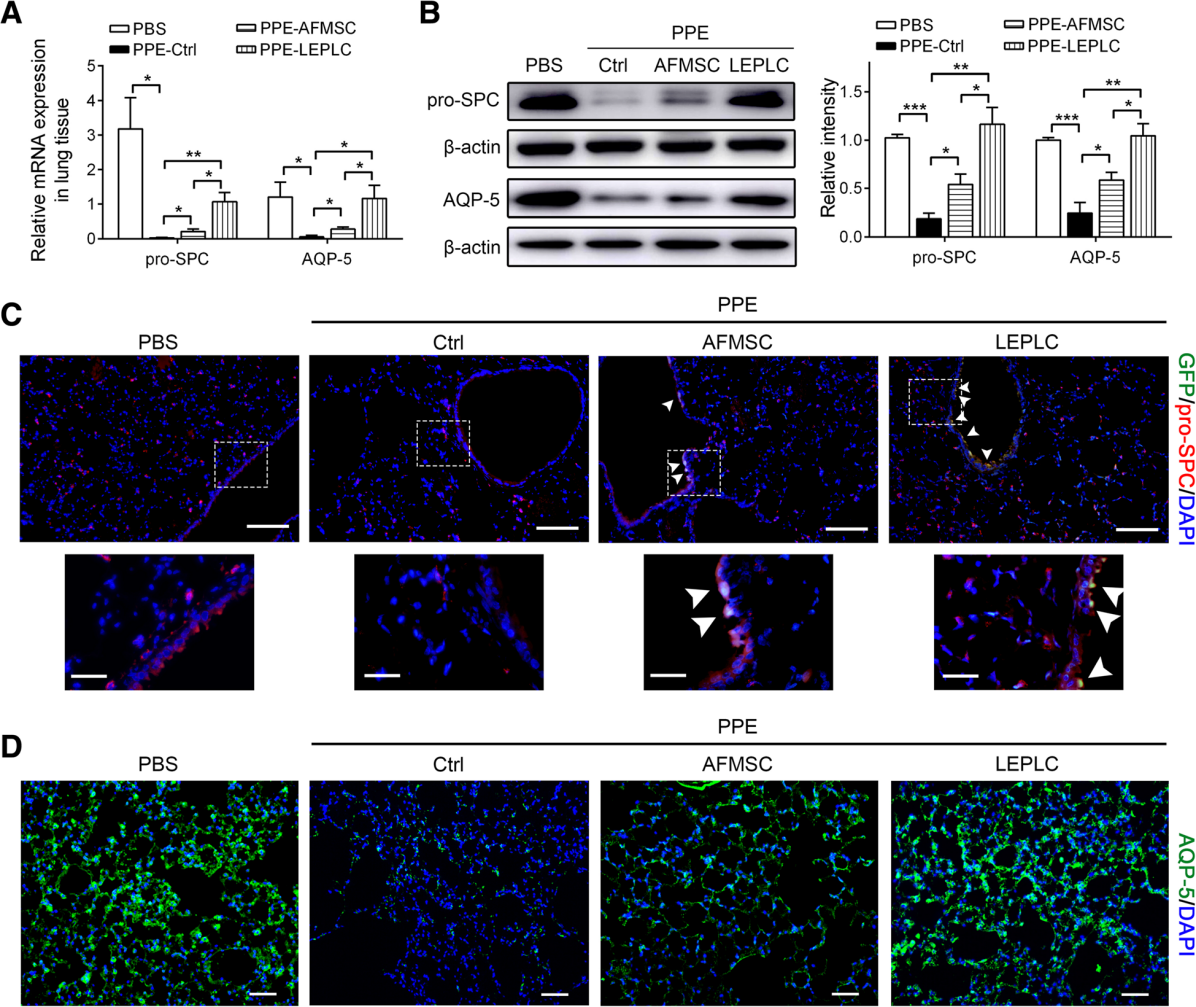
Transplantation of LEPLCs enhanced the expression of lung epithelial progenitor-like markers in the PPE-induced pulmonary emphysema mouse model. **a** Quantitative real-time RT-PCR and **b** Western blots analysis expression of SPC and AQP-5 in whole lung tissues of animals that received PBS, AFMSCs, or LEPLCs after 4-week period of PBS, AFMSCs, or LEPLCs treatment following PPE administration. Values were normalized to the *Gapdh* values and were expressed relative to the PBS group. The histogram on the right shows the semiquantitative densitometry of the Western blot analysis determined using ImageJ. Data are expressed as the mean ± SD; *n* ≥ 5 per group. *p < 0.05, **p < 0.01, and ***p < 0.001. **c**, **d** Immunofluorescence staining of (**c**) GFP-positive cells (green) and type II alveolar epithelial markers, pro-SPC (red); **d** type I alveolar epithelial markers, AQP-5 (green), and nucleus (DAPI; blue) in mouse lung tissues from each group treated as described above. The white dotted line areas in the (**c**) upper panel are magnified in the lower panel with 10 μm bars, and the expressions of pro-SPC and GFP were co-localized (yellow, arrowheads). Scale bar = 100 μm

**Fig. 7:**
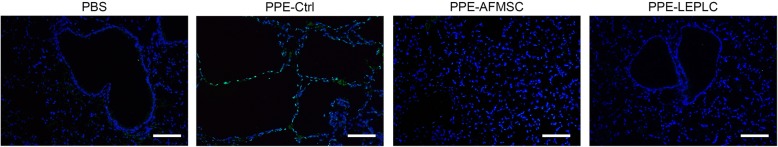
Transplantation of LEPLCs attenuated apoptosis in the PPE-induced pulmonary emphysema mouse model. C57BL/6 mice received PBS, AFMSCs, or LEPLCs after 4-week period of PBS, AFMSCs, or LEPLCs treatment following PPE administration. Lung tissues were subjected to immunofluorescence staining to determine the colocalization of apoptotic cells (TUNEL stain; green) and nucleus (DAPI; blue). Scale bar = 100 μm

Impairment of alveolar regeneration and increased apoptosis in structural alveolar cells contribute to emphysema [5]. After PPE treatment, the mRNA and protein expression levels of the type I and type II alveolar epithelial cell-specific markers (AQP-5, and SPC) were markedly reduced when compared with the PBS control (Fig. 6a and b). Transplantation of AFMSCs increased the expression levels of SPC and AQP-5, which were further increased in the LEPLC-transplanted mice (Fig. 6a and b). Immunofluorescence staining also observed that the number of SPC-positive and AQP-5-positive cells in the lung tissue was obviously reduced 6 weeks after post-PPE treatment. Transplantation of either AFMSCs or LEPLCs recovered the number of SPC-positive and AQP-5-positive cells in the damaged lung tissue (Fig. 6c and d). In addition, part of the SPC-positive cells was specifically distributed around the bronchi that expressed GFP protein (Fig. 6c and d). After 6 weeks post-PPE treatment, the number of TUNEL-positive cells significantly increased, indicating the presence of more apoptotic cells within the lung alveolar wall. Transplantation of either AFMSCs or LEPLCs significantly reduced the incidence of PPE-induced apoptotic cells in the lung tissue (Fig. 7).

Taken together, our data indicated that transplanted LEPLCs engrafted, attenuated inflammatory and fibrotic effects, increased alveolar regeneration, and reduced structural alveolar cell apoptosis in PPE-induced pulmonary emphysema mice.

## Discussion

This is the first report using predifferentiated AFMSCs to treat mouse lung emphysema. Because of the low efficiency of accurate differentiation at lung damaged site and the heterogeneity of undifferentiated MSCs, these are unsuitable for clinical application. Thus, the predifferentiated strategies not only could enrich the lung progenitors to accelerate and reconstruct the damaged area, but also be great help in developing minimally self-originating regenerative therapeutic.

The use of PPE to induce experimental emphysema, where lung injury develops rapidly, easily, inexpensively, and severely than cigarette smoke [40]. In the present study, we used a model of a single intratracheal instillation of PPE, which causes histological and morphological characteristics, lung inflammation, and collagen deposition closely resembling human emphysema [41]. MSCs were administered 2 weeks after the PPE administrate, when alveolar structure was destructed, and mild emphysema were already established [12].

Following the promising results of MSC-based cell therapy in an experimental model of pulmonary emphysema in 2006 [42], a variety of sources of MSCs (bone marrow, adipose tissue, lung, amniotic fluid) have been transplanted into proteolytic enzyme- or cigarette smoking-induced COPD-like animal models. These studies have shown a therapeutic potential for transplanted MSCs on improving lung architecture, lung tissue remodeling, modulating inflammatory response, and decreasing apoptosis and collagen accumulation in pulmonary emphysema mouse model [43–45]. In this study, we used amniotic fluid mesenchymal stem cells, which possess self-renewal capacity, clonal properties [28], and multilineage differentiation ability (Additional file 2: Figure S1), and lack teratoma formation potential and ethical concerns [46]. In comparison with that of Li et al. in which AFMSC-based therapy can alleviate the lung emphysema [16], our results further observed that transplanted AFMSCs had yielded beneficial effects on reducing inflammation and fibrosis, enhancing lung alveolar epithelium regeneration and reversing emphysema.

The low efficiency of accurate and precise differentiation of engrafted stem cells in the damaged lung reinforces the notion that progenitor cell populations are considered as better therapeutic agents for accelerate healing and reconstruct the damaged area [47]. In addition, CCSP-positive Clara cells are thought as a progenitor cell population for the distal conducting airways and could theoretically contribute to alveolar epithelial repair in naphthalene-injured rodent model [48]. Other evidence observed that β4 integrin-expressing AEC-progenitor or p63/keratin 5-expressing basal epithelial-progenitor populations may play a role in alveolar epithelial regeneration in fibrotic lung [49]. However, a lung progenitor cell showed benefit on directly differentiating to specific cellular lineage, but studies mentioned that progenitor cell cannot be maintained in a proliferative state over multiple passage. Therefore, generating a pure and abundant population of lung progenitor cells is a key problem [50]. Taken together, transplantation of undifferentiated cells and progenitors had both therapeutic benefit and limitation; we therefore see value in using predifferentiated MSCs into alveolar epithelial lineage prior to transplant in the emphysema model.

Based on this idea, Wang et al. established a stable genetic modified hESCs that can be differentiated and enriched into a pure population of type II alveolar epithelial cells may be suitable for transplantation to treat lung injury [23]. Other reports have established predifferentiating protocol for ESCs [51] or iPSCs [24, 52] into alveolar epithelial cells, and transplantation of these predifferentiated cells exerts a better therapeutic approach in lung disease models.

Mounting evidence has shown that transplanted MSCs exert their beneficial effects in part via paracrine factors and/or transdifferentiation to epithelial cells that contribute to restoring the destructed lung architecture [26, 44]. In addition, evidence showed that MSC-based tissue repair partly relies on secreting paracrine factors to establish a regenerative microenvironment and repair the damaged sites [53]. The potential mechanism of transplanted AFMSCs may act by modulating inflammation, apoptosis, fibrosis, and cell proliferation via the paracrine effectors [54, 55]. We further evaluated the therapeutic potential of these predifferentiated AFMSCs in a PPE-induced lung emphysema mouse model. Reports showed that both type I and II alveolar epithelial cells are constantly contributing to airway defense. Specifically, type II alveolar epithelial cells may secrete a variety of mediators to modulate the inflammatory response in lung injury [56, 57]. In this study, we observed downregulation of the expression of inflammatory factors, pro-IL-β, IL-6, INOS, and MCP-1 upon transplantation of either AFMSCs or LEPLCs. Both transplantation groups showed improvement in terms in the presence of fibrosis, and both groups had increased SPC^+^ (type II alveolar epithelial cell) and AQP-5^+^ (type I alveolar epithelial cell) cell regeneration.

Previous studies have shown that transplanted stem cells via intratracheal injection may distribute around the bronchi [32, 35] and may be found in alveolar capillaries or larger blood vessels after an intravenous injection [58]. Our results also observed that transplanted AFMSCs or LEPLCs were engrafted surrounding the bronchi. Thus, we propose that transplantation of either AFMSCs or LEPLCs may restore damaged type II alveolar epithelial cell around the bronchi and repair the alveoli structure collapse which heavily relies on paracrine effects to establish a regenerative microenvironment eventually repair the damaged sites.

Cumulative observations found that coexistence emphysema (ECM breakdown) and fibrosis (ECM overaccumulation) should not be that unusual [59], which is in line with the increased and accumulated collagen as observed in airways of our PPE-induced emphysema mouse model. One possible mechanism is that elastase degradation relative abundance of collagen and elastin leads to alveolar destruction. At the same time, elastase may promote myofibroblast differentiation at the regions of alveolar wall destructive tissue to restore collagen content, eventually causing fibrosis [60]. In the remodeling process of emphysema, TGF-β plays a key role in stimulating myofibroblast proliferation and secretion of collagen fibers [61]. Either AFMSCs or LEPLCs reduced collagen accumulation in airways and TGF-β1 expression level; hence, LEPLCs were able to completely restore collagen level in the lung parenchyma.

## Conclusion

This study uses an optimal differentiation condition of mouse amniotic fluid mesenchymal stem cells towards LEPLCs in vitro under modified small airway growth medium (mSAGM). Transplantation of pre-differentiated LEPLCs through intratracheal injection adapted to the microenvironment, attenuated inflammatory and fibrotic effects, recovered alveolar regeneration, and reduced structural alveolar cells apoptosis in PPE-induced pulmonary emphysema mice.

## Additional files

Additional file 1: **Table S1.** Primer sequences. (DOCX 19 kb)

Additional file 2: **Figure S1.** Characterization of amniotic fluid mesenchymal stem cells (AFMSCs) isolated from eGFP-expressing transgenic mice. (A and B) The morphologies of identified mouse AFMSCs in single layer under bright and fluorescence fields, respectively. (C and D) Differentiation of AFMSCs into mesodermal cell types after specific induction for 21 days is marked by the appearance of lipid granules (adipogenic) by Oil Red O staining and mineralized matrix (osteogenic) by Alizarin red staining. Scale bar = 200 μm. (E) Immunophenotypes of eGFP-AFMSCs by flow cytometric analysis of the cell surface antigens CD44, Sca-1, CD105, CD34, CD29, CD11b, CD90, CD73, CD106, and CD45, respectively. (JPG 6883 kb)

Additional file 3: **Figure S2.** Predifferentiation of AFMSCs in modified small airway growth medium (mSAGM) for 5- and 9-day induction. AFMSCs were differentiated for 5- and 9-days and then immunostained for lung epithelial progenitor-like markers, TTF-1, SPC, AQP-5, and CCSP. Flow cytometry analysis was performed. (JPG 556 kb)

## Data Availability

Not applicable.

## Abbreviations

AFMSCs: Amniotic fluid mesenchymal stem cells
AQP-5: Aquaporin 5
CCSP: Clara cell secretory protein
COPD: Chronic obstructive pulmonary disease
ICAM-1: Intercellular adhesion molecule 1
iNOS: Inducible nitric oxide synthase
LEPLCs: Lung epithelial progenitor-like cells
MCP-1: Monocyte chemotactic protein-1
MMP-9: Matrix metalloproteinase-9
mSAGM: Modified small airway growth medium
PPE: Porcine pancreatic elastase
SABM: Small airway basal medium
SPC: Surfactant protein C
TGF-β1: Transforming growth factor-beta 1
TTF-1: Thyroid transcription factor 1
TUNEL: Terminal deoxynucleotidyltransferase-mediated dUTP nick-end labeling
VEGF: Vascular endothelial growth factor
