# Supplementary material for: Predifferentiated amniotic fluid mesenchymal stem cells enhance lung alveolar epithelium regeneration and reverse elastase-induced pulmonary emphysema
Source: Stem Cell Res Ther. 2019 Jun 13;10:163. doi: 10.1186/s13287-019-1282-1 (PMC6567664; doi:10.1186/s13287-019-1282-1)
Supplement: Supplementary file 1 — Table S1. Primer sequences. (DOCX 19 kb) [file 13287_2019_1282_MOESM1_ESM.docx]

**Supporting Information Table**

**Table S1. Primer Sequences**

| Gene | Primer Sequence | Tm(^o^C) | Product Size (bp) |
| --- | --- | --- | --- |
| GFP | Forward: ACCGGGGTGGTGCCCATCCT | 60 | 314 |
|  | Reverse: TTCACCTCGGCGCGGGTCTT | 58 |  |
| Pro-IL-1β | Forward: GCTCATCTGGGATCCTCTCC | 56 | 242 |
|  | Reverse: CCTGCCTGAAGCTCTTGTTG | 54 |  |
| IL-6 | Forward: CCACTTCACAAGTCGGAGGCTTA | 57 | 112 |
|  | Reverse: GCAAGTGCATCATCGTTGTTCATAC | 56 |  |
| iNOS | Forward: CACCTTGGAGTTCACCCAGT | 54 | 170 |
|  | Reverse: ACCACTCGTACTTGGGATGC | 54 |  |
| MCP-1 | Forward: TCACTGAAGCCAGCTCTCTCT | 54 | 127 |
|  | Reverse: GTGGGGCGTTAACTGCAT | 50 |  |
| Col I | Forward: CTGACTGGAAGAGCGGAGAGTAC | 59 | 121 |
|  | Reverse: ACAGACGGCTGAGTAGGGAACA | 57 |  |
| Col III | Forward: GTTCTAGAGGATGGCTGTACTAAACACA | 58 | 76 |
|  | Reverse: TTGCCTTGCGTGTTTGATATTC | 51 |  |
| TGF-β1 | Forward: TTGCTTCAGCTCCACAGAGA  Reverse: TGGTTGTACAGGGCAAGGAC | 52 | 183 |
|  |  | 54 |  |
| elastin | Forward: GCTGGAGGTTTAGTGCCTGG | 56 | 200 |
|  | Reverse: GCTCCGTATTTGGCAGCTTT | 52 |  |
| ICAM-1 | Forward: CAGCAGACTCTGAAATGCCAG | 54 | 201 |
|  | Reverse: TTGAGAGTGGTACAGTACTGTCAG | 56 |  |
| MMP9 | Forward: CTGGACAGCCAGACACTAAAG | 54 | 145 |
|  | Reverse: CTCGCGGCAAGTCTTCAGAG | 56 |  |
| VEGFa | Forward: CTGTGCAGGCTGCTGTAAC | 53 | 184 |
|  | Reverse: ACAGTGATTTTCTGGCTTTGTTC | 52 |  |
| SPC | Forward: ATGGACATGAGTAGCAAAGAGGT | 53 | 117 |
|  | Reverse: CACGATGAGAAGGCGTTTGAG | 54 |  |
| SPA | Forward: CAGTGTGATTGGGAGAAACC | 52 | 297 |
|  | Reverse: TGTCTCCATGTTCTCCAGGT | 52 |  |
| AQP5 | Forward: ATCTACTTCACCGGCTGTTCC | 54 | 260 |
|  | Reverse: GTCAGCTCGATGGTCTTCTTC | 54 |  |
| GAPDH | Forward: TGACCTCAACTACATGGTCTACA | 53 | 85 |
|  | Reverse: CTTCCCATTCTCGGCCTTG | 53 |  |

GFP, green fluorescent protein; IL6, interleukin 6; iNOS, inducible nitric oxide synthase; MCP-1, monocyte chemoattractant protein-1; Col I, collagen type I; TGF-β1, transforming growth factor beta 1; MMP9, matrix metallopeptidase 9; VEGFa, vascular endothelial growth factor a; SPC, surfactant C; AQP5, aquaporin 5; GAPDH, glyceraldehyde-3-phosphate dehydrogenase.
