# Supplementary figures and images for: Predifferentiated amniotic fluid mesenchymal stem cells enhance lung alveolar epithelium regeneration and reverse elastase-induced pulmonary emphysema
Source: Stem Cell Res Ther. 2019 Jun 13;10:163. doi: 10.1186/s13287-019-1282-1 (PMC6567664; doi:10.1186/s13287-019-1282-1)

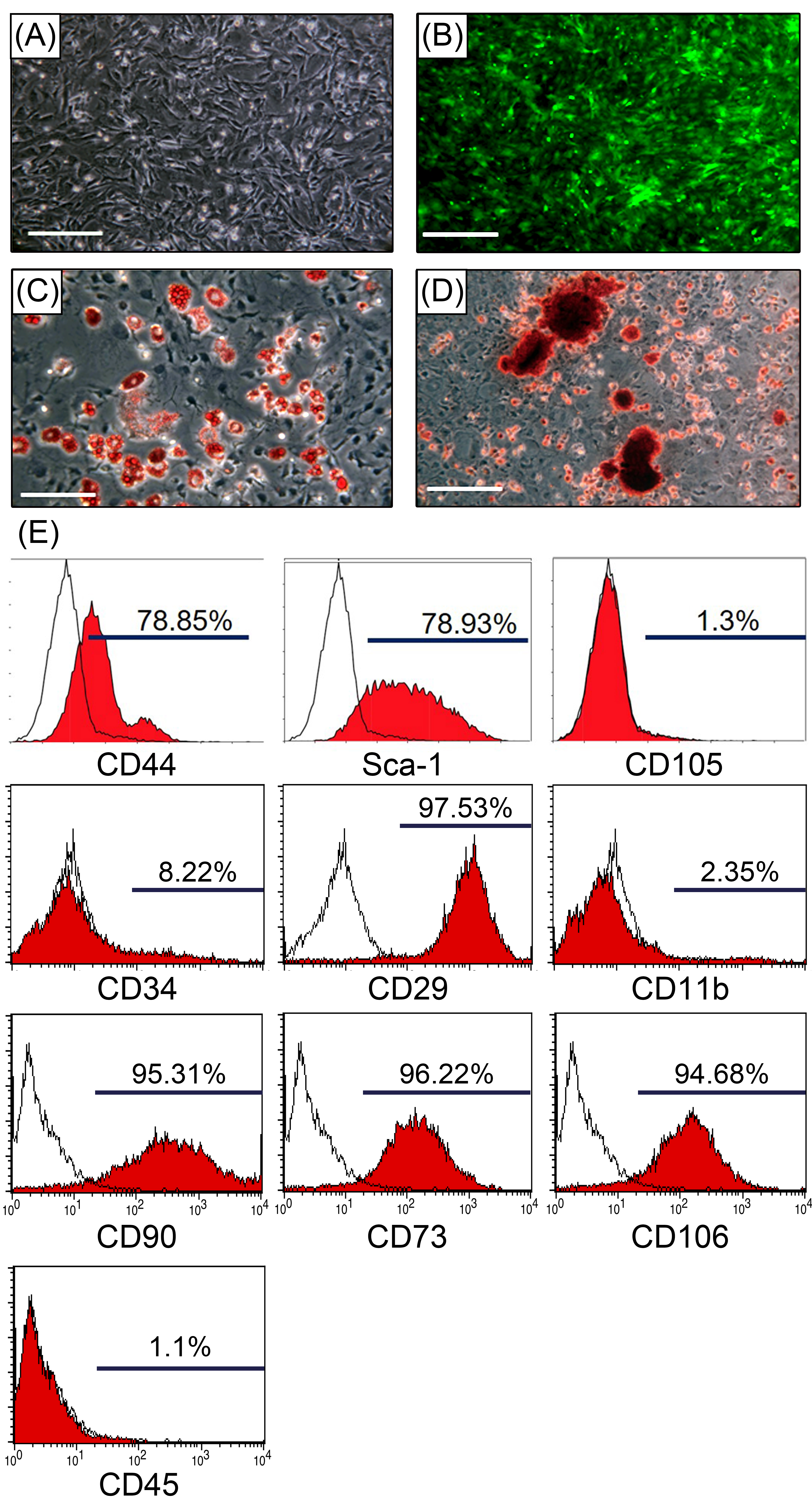

Supplement: Supplementary file 2 — Figure S1. Characterization of amniotic fluid mesenchymal stem cells (AFMSCs) isolated from eGFP-expressing transgenic mice. (A and B) The morphologies of identified mouse AFMSCs in single layer under bright and fluorescence fields, respectively. (C and D) Differentiation of AFMSCs into mesodermal cell types after specific induction for 21 days is marked by the appearance of lipid granules (adipogenic) by Oil Red O staining and mineralized matrix (osteogenic) by Alizarin red staining. Scale bar = 200 μm. (E) Immunophenotypes of eGFP-AFMSCs by flow cytometric analysis of the cell surface antigens CD44, Sca-1, CD105, CD34, CD29, CD11b, CD90, CD73, CD106, and CD45, respectively. (JPG 6883 kb) [file 13287_2019_1282_MOESM2_ESM.jpg]

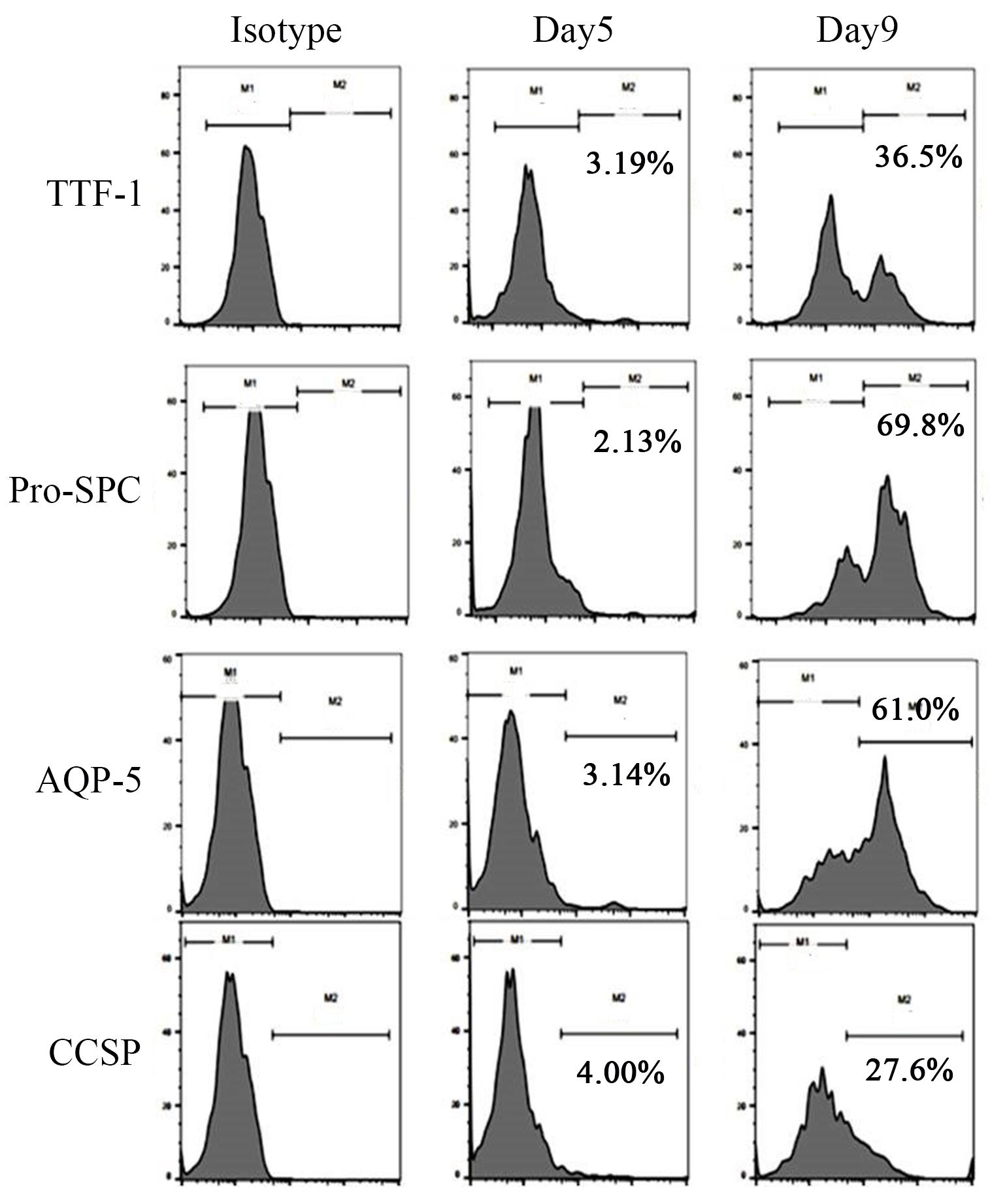

Supplement: Supplementary file 3 — Figure S2. Predifferentiation of AFMSCs in modified small airway growth medium (mSAGM) for 5- and 9-day induction. AFMSCs were differentiated for 5- and 9-days and then immunostained for lung epithelial progenitor-like markers, TTF-1, SPC, AQP-5, and CCSP. Flow cytometry analysis was performed. (JPG 556 kb) [file 13287_2019_1282_MOESM3_ESM.jpg]
